# The Effects of Experimental Sleep Apnea on Cardiac and Respiratory Functions in 6 and 18 Month Old Dystrophic (*mdx*) Mice

**DOI:** 10.1371/journal.pone.0147640

**Published:** 2016-01-25

**Authors:** Milind R. Chaudhari, James A. Fallavollita, Gaspar A. Farkas

**Affiliations:** 1 Department of Exercise and Nutrition Sciences, University at Buffalo, Buffalo, New York, United States of America; 2 Department of Medicine, University at Buffalo, Buffalo, New York, United States of America; 3 Department of Epidemiology and Environmental Health, University at Buffalo, Buffalo, New York, United States of America; 4 Center for Research in Cardiovascular Medicine, University at Buffalo, Buffalo, New York, United States of America; 5 VA Western New York Healthcare System at Buffalo, Buffalo, New York, United States of America; Rutgers University -New Jersey Medical School, UNITED STATES

## Abstract

Duchenne muscular dystrophy (DMD) is a fatal disease where over 90% of patients succumb to respiratory or cardiac failure. Sleep apnea and sleep disordered breathing (SDB) are noted in a plurality of DMD patients, and the resulting nocturnal episodic hypoxia (EH) cannot be ruled out as a contributing factor to cardiac and respiratory dysfunction. In this study, we investigated the impact of long-term episodic hypoxia, which mimics the cyclic hypoxia seen in sleep apnea, on cardiac and respiratory function in a murine model of DMD (*mdx* mice). Since the severity and prevalence of sleep apnea in DMD increases with age, we studied the impact of EH on young (6-month) and on older (18-month) *mdx* mice. Mice were either exposed for 12 weeks to EH (8 hours/day, 5 days/week) or to room air. We noted a significant increase in left ventricular (LV) dilatation (transthoracic echocardiography) on EH exposure in both age groups, but reduced LV contractility was seen only in 6-month old mice. With EH exposure, an increased fibrosis (hydroxyproline) was noted in both cardiac and diaphragm muscle in 18-month but not 6-month old mice. No significant change in relative diaphragm strength (in-vitro) was noted on EH exposure in 18-month old mice. In contrast, EH exposed 6-month old mice showed a significant increase in relative diaphragm strength. EH exposure did not result in any significant change in ventilatory parameters (barometric plethysmography) in awake 6-month old *mdx* mice. In contrast, 18-month old *mdx* mice showed considerable ventilatory dysfunction, consistent with reduced ventilatory reserve. Our findings highlight that sleep apnea impacts respiratory and cardiac function in muscular dystrophy, and that EH can have divergent effects on both systems. To our knowledge, this is the first comprehensive study to investigate the impact of EH on cardiac and respiratory function in *mdx* mice.

## Introduction

Duchenne muscular dystrophy (DMD) is an X-linked recessive neuromuscular disorder that affects approximately 1 out of 3500 male births [1]. DMD is a lethal disease that is characterized by the absence of dystrophin, a structural protein in skeletal and cardiac muscle, that links the cytoskeleton to the extracellular matrix [2, 3]. Dystrophin deficient skeletal and cardiac muscles, are susceptible to a progressive myopathy. Indeed, respiratory and cardiac muscle dysfunction together contributes to the death of over 90% of DMD patients [3, 4].

Ventilatory abnormalities and related respiratory symptoms are noted early in the course of DMD. Respiratory muscle weakness, especially that of the diaphragm, leads to respiratory insufficiency and 2/3 of DMD patients die in their third decade from respiratory failure [4]. By 18 years of age, cardiac manifestations are also detected in about 90% of DMD patients. The most prevalent cardiac defect in DMD is dilated cardiomyopathy, and eventually leads to cardiac failure in 10–20% of DMD patients [4].

Sleep is an especially vulnerable period for patients with DMD, and many are diagnosed with sleep disordered breathing (SDB). It has been reported that >80% DMD patients have SDB with overt signs of alveolar hypoventilation [5, 6]. Smith et al. [7] reported that patients with DMD endure bouts of episodic hypoxia (EH) during sleep, during which SaO_2_ levels fall from baseline values of 95.4 ± 0.6% to a mean nadir of 74.2 ± 3.9%. These nocturnal episodes of hypoxemia were reported to be more severe and more frequent in older DMD patients. Indeed, sleep apnea in non-DMD patients is known to contribute to cardiovascular complications such as systemic hypertension, coronary heart diseases and stroke [8–10]. Due to the high incidence of SDB in DMD patients, we hypothesized that EH may be a contributing factor to their cardiac and respiratory dysfunction.

The *mdx* mouse, a murine model of DMD presenting with a deficiency of dystrophin, results in a progressive myopathy of the diaphragm [11, 12]. Cardiomyopathy is also observed in *mdx* mice, but at a later age. Signs of cardiomyopathy are detected in *mdx* mice at 9–10 months of age and become more prominent with age [13, 14].

Although cardiac and respiratory dysfunctions consistent with DMD are indeed detected in *mdx* mice, they do not have spontaneous SDB. In order to better define the possible consequences of SDB that is so prevalent in DMD, the current study assessed the putative impact of EH on cardiac and respiratory function in *mdx* mice. EH is a standard and well-studied technique for experimental sleep apnea in mice [15]. Since SDB is more severe and prevalent in older DMD patients, we investigated whether the effects of EH in *mdx* mice are compounded by age. The purpose of this study, therefore, was to assess the impact of EH on cardiac and respiratory function in 6 and 18-month old *mdx* mice. To our knowledge, this is the first comprehensive study to investigate the consequences of EH simultaneously on cardiac and respiratory function in young and older *mdx* mice.

## Methods

The study was performed on 80 male *mdx* mice (C57BL/10ScSn-*Dmd*^*mdx*^/J) purchased from Jackson Laboratories (Maine, USA). The animals were purchased at 3–4 weeks of age and housed in the Laboratory Animal Facility at the University at Buffalo. Mice of similar age were housed five to six per cage with food and water available *ad libitum*. Throughout the study, mice were maintained on a 12 hour light/dark cycles (6:00 am-6:00 pm).

### Ethics Statement

All the experimental protocols were approved by the Institutional Animal Care and Use Committee (IACUC) of the University at Buffalo (Permit Number: NUT12530N). Tissue collection was performed under anesthesia (ketamine/xylazine) in order to minimize pain and distress.

### Episodic Hypoxia (EH)

Starting at 3-mo of age and at 15-mo of age, 40 *mdx* mice from each age group were randomly assigned either: 1) to a control group (n = 20) exposed continuously to room air (RA) or 2) to an EH group (n = 20) exposed 5 days a week, 8 hours per day for 12 weeks to EH. Mice were weighed weekly throughout the experimental period. Studies in young and old mice were run concurrently. Mice assigned to the EH protocol were slowly habituated over a 2 weeks period to progressively longer and more severe levels of hypoxia. This initial habituation period was not included in the 12 week experimental period. Mice of both age groups assigned to the EH group were placed 8 hours a day during the diurnal period in sealed chambers (8:00 am and 4:00 pm, the normal sleep period for mice). During EH exposure, the O_2_ levels in the chamber were altered repeatedly in cycles (90-second cycle length) between 21% and 6% by alternating the flow of N_2_ (99.5%, medical grade) and room air. The O_2_ levels in the cages were continuously measured with an oxygen analyzer (S-3A, AEI technology Inc. Pittsburgh, PA). Mice housed within a given cage were all treated to the same exposure (EH or RA). Mice were returned to their home cages at the conclusion of the daily EH exposure. RA and EH mice were housed in a common room, and were thus exposed to the same environmental conditions (sounds from valves opening/closing). Food and water were available *ad libitum* during all phases of the study.

At the end of the 12 week experimental period, mice from all four groups were studied in a random order. The younger mice were designated as 6-mo RA or 6-mo EH, while the older mice were designated as 18-mo RA or 18-mo EH.

### Respiratory function analysis

**Fig 1:**
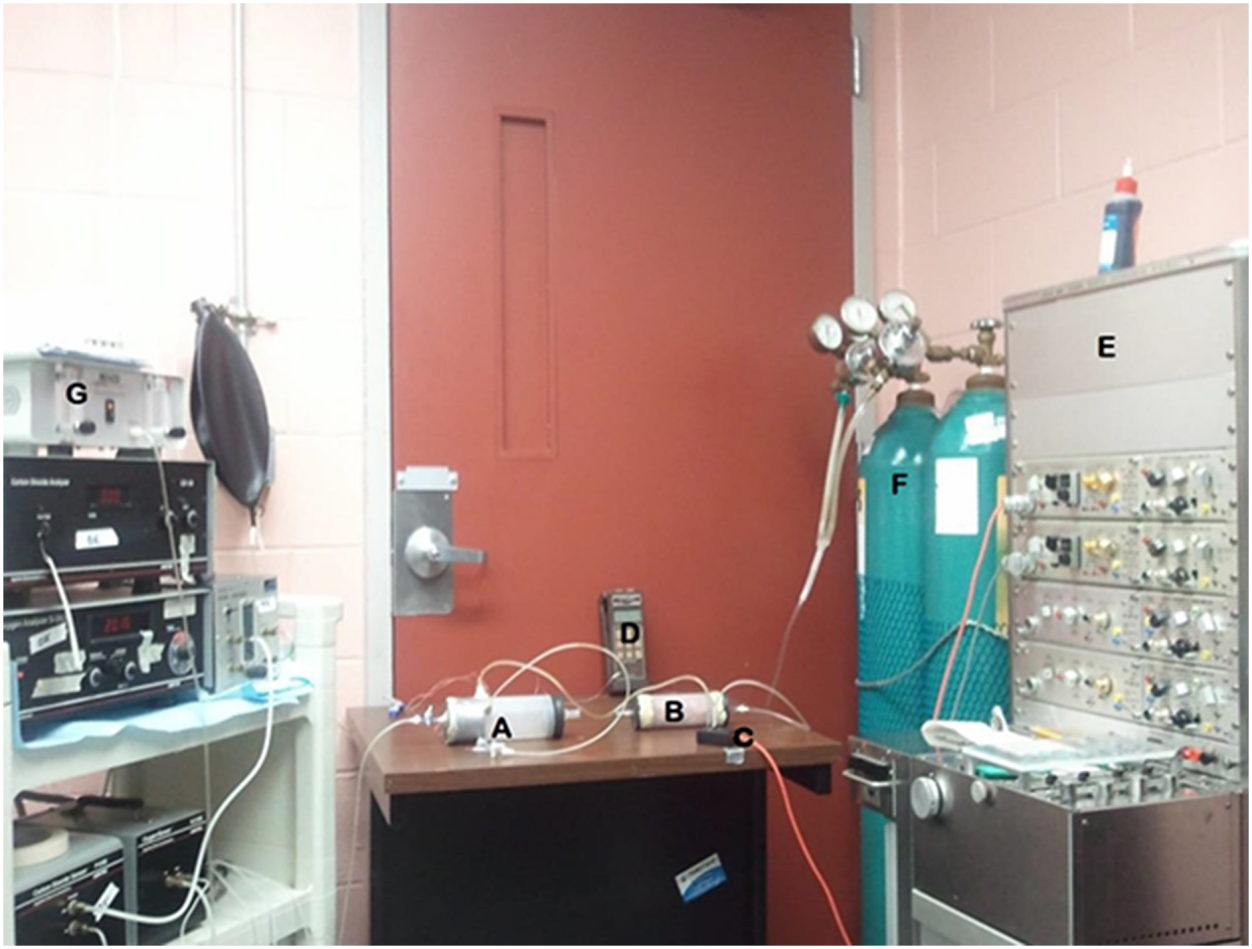
Modified Barometric Plethysmography. Photograph of plethysmography set up for ventilatory function analysis. (A) Animal chamber; (B) Reference chamber; (C) Transducer; (D) Thermometer; (E) Polygraph machine; (F) Gas mixture; (G) Flow meter.

In the 2 weeks prior to the terminal procedures, and over the weekend as not to interfere with the daily exposure to EH, breathing patterns in awake mice were measured using a modification of the whole body plethysmography technique [16]. Mice were placed inside a chamber that prevented backward rotation but did not cause chest wall compression. Mice were habituated on numerous occasions to a restrainer as well as to plethysmography in order to minimize any effects of stress related changes in ventilation. The apparatus was connected to a flow meter (Dwyer Instruments Inc., Michigan City, IN), a pressure transducer (Model #7, Hewlett Packard, Palo Alto, CA) and a polygraph machine (Model 7 polygraph, Grass, Quincy, MA) for the purpose of maintaining a constant air flow, detecting pressure changes due to breathing and recording the transducer signal, respectively. The inflowing and outflowing O_2_ concentrations were measured by O_2_ analyzer (S-3A, AEI technology Inc. Pittsburgh, PA) (Fig 1).

Ventilation was measured at rest and during hypercapnic respiratory stimuli (4% CO_2_, 26% O_2_, and Bal N_2_). Resting ventilation was measured 15 min after placing the animal in the chamber and 5 minutes after administering the hypercapnic mixture. To record ventilation, airflow was momentarily stopped and the chamber was sealed after which pressure changes due to breathing were recorded. An amount of 0.1 mL was injected and withdrawn via a 1 cc syringe into the chamber for the purpose of calibration.

Body temperatures of all mice were assumed to be 37˚C and to remain constant during the ventilation protocol. Chamber temperature was monitored continuously via a probe mounted inside the chamber. Barometric pressure measurements were recorded every day. Breathing frequency (f) was calculated directly from the ventilation-induced pressure swings. Tidal volume (Vt) was measured as a function of the pressure change inside the chamber, using an equation by Drorbaugh and Fenn [17] modified by Bartlett and Tenney [18]. Respiratory ventilation (Ve) was calculated (Ve = Vt × f) and values were expressed per body weight (ml/kg/min).

### Echocardiography

Transthoracic echocardiography was performed on anesthetized [ketamine (100mg/kg) -xylazine (5 mg/kg) IP] mice using Vivid 7 echo machine (GE medical systems) with a 13-MHz linear transducer (i13L). Mice were held in the supine position, the anterior chest wall was shaved, warm ultrasound gel applied to the chest, and the transducer probe was placed over the left hemithorax. Parasternal and short-axis two-dimensional images of the left ventricle (LV) were obtained to determine correct M-mode cursor positioning. Multiple short axis M-mode images of the left ventricle were obtained and these images were analyzed for LV functional parameters in triplicate. Heart rate (HR) was determined from at least three consecutive RR intervals. The other LV indices measured included LV internal dimension at end-diastole (LVIDd), and end-systole (LVIDs), septal wall thickness (SWT) and posterior wall thickness (PWT) as recommended by the American Society of Echocardiography[19]. Left ventricular end-systolic volume (ESV) and end-diastolic volume (EDV) were estimated assuming spherical geometry. The LV percent fractional shortening (%FS) was calculated as follows: %FS = 100 x (LVIDd–LVIDs) / LVIDd. Estimated LV mass (in diastole) was calculated from following formula: LV mass (g) = 0.80 x [1.04 (IVSd + LVPWd + LVIDd) ^3^ - (LVIDd)^3^] + 0.6. All the LV dimension data are presented as the average of measurements from at least 3 selected beats on the image.

### Tissue removal and blood collection

Following completion of the echocardiography, blood was collected from the orbital vein in a capillary tube for hematocrit (Hct) analysis.

The diaphragm (costal and crural) muscle was then quickly removed and placed in cooled (4°C) Kreb’s solution aerated with a 95% O_2_, 5% CO_2_ gas. The diaphragm was cut into two halves, each containing both crural and costal parts. One half was used for measurement of contractile properties while the other half was either frozen in liquid N_2_ for biochemical properties or mounted on thick cork sheet, supported with O.C.T. compound and snap-frozen in isopentane (2-methylbutane) cooled to its melting point with liquid N_2_ for histological analyses. Muscle tissue samples were then stored at -80°C for subsequent histological or hydroxyproline analysis.

The whole heart was then removed, blotted dry on filter paper and weighed. The heart was then either cut horizontally through the ventricles and mounted on thick cork sheet, supported with O.C.T. compound, and snap-frozen in isopentane (2-methylbutane) cooled to its melting point with liquid N_2_ for histology or frozen in liquid N_2_ and stored at -80°C for subsequent hydroxyproline analysis.

### Contractile properties of diaphragm

Details of the technique employed for measuring *in vitro* muscle contractile properties have been described previously [20]. In brief, portions of the costal diaphragm muscle were removed, cut into small bundles, and placed in a muscle bath. The muscle bath was filled with Krebs solution composed of the following (mM): NaCl, 137; KCl, 4; MgCl_2_, 1; KH_2_PO_4_, 1; NaHCO_3_, 12; CaCl_2_, 2; and glucose, 6.5. The diaphragm segments were vertically mounted between large platinum stimulating electrodes and immersed in a Krebs solution maintained at 36°C perfused with 95% O_2_-5% CO_2_. The muscle bundles were stimulated supra-maximally with monophasic pulses of 0.2 ms duration.

Isometric force measurements were recorded with a Grass FT-03C force transducer (Grass Instruments, Quincy, Massachusetts). Muscle force signals were displayed on an oscilloscope and measured directly from the oscilloscope tracings. Muscle length was changed by raising or lowering the force transducer to a desired level. After a 5-min thermo-equilibration period, the bundles were adjusted to L_o_, defined as the length at which peak twitch force were recorded. L_o_ was measured in triplicate using a micrometer. The following procedures were performed in the following order: (1) twitch characteristics, (2) force-frequency response.

#### Twitch Characteristics

In each muscle bundle, five twitches were recorded at L_o_ and the average value was used to determine time-to-peak tension (TPT), half-relaxation time (½RT), and twitch force (P_t_). TPT is defined as the time from the start of the contraction to the point where peak tension was achieved. ½RT is defined as the time from P_t_ to the point where twitch tension dropped by 50%.

#### Force-Frequency Curve

Each muscle bundle was sequentially stimulated at frequencies of 10, 20, 35, 50, 70, 100, 120, and 150 Hz. The stimulation was maintained until plateau in force was attained, usually requiring 350–500 ms. Approximately 30 sec elapses between each stimulus train. Maximum tetanic force (P_o_) was recorded and the twitch-to-tetanus ratio (P_t_/P_o_) was calculated for each muscle bundle.

On completion of the measurements, the bundle was removed from the apparatus, blotted dry, and weighed. Muscle cross-sectional area (CSA) was approximated by dividing the muscle mass by its length and muscle density (1.056 g/cm^3^) [21]. Specific force was expressed as N/cm^2^.

### Histological analysis

Tissue samples previously mounted on cork for histological analysis were removed from the freezer and equilibrated to -20°C for 15–20 min. The cork was mounted on a Microtome cryostat block with O.C.T. compound. Serial sections (10 μ thick) of the tissue were cut, mounted on glass cover slides and stained by H&E method [22].

Images of the tissues (10x, 20x) were obtained with a color coolSNAP CCD camera and analyzed with Metamorph imaging software (Molecular Devices, CA, USA). Tissue slides and images were coded and the individual performing the analysis was blinded to the code. For each muscle tissue, at least 200 myofibers were analyzed for fiber size, number of central nuclei, tissue thickness and percent area of interstitial space [23].

### Hydroxyproline analysis

To determine collagen content, a whole heart and a total hemi-diaphragm were assayed for hydroxyproline. A modified colorimetric hydroxyproline assay adapted from Prockop and Udenfriend [24] and Switzer and Summer [25] was used. For diaphragm, the tissue was dry blotted, weighed and then hydrolyzed using 6 N HCl (1ml HCl per 10mg of tissue) at 110°C for 16 hours. For determination of heart hydroxyproline content, whole heart tissue was dry blotted, weighed and then hydrolyzed using 6N HCl (0.5ml HCl per 10mg of tissue) at 120°C for 48 hours. Samples of hydrolysate from either tissue were then diluted with deionized water and oxidized using chloramine-T solution for 25 minutes. Toluene and KCl was added to remove the impurities. The remaining solution was heated in boiling water for 30 minutes. Toluene extraction was repeated again and Ehrlich’s reagent (*p*-dimethylamino-benzaldehyde) was mixed with the toluene layer. After 30 minutes of incubation, the diaphragm and heart tissue samples were read using a BioTek microplate reader (BioTek Instruments, Winooski, VT) against hydroxyproline standards (0.75, 1.5, 3.0, 6.0 μg) at 562nm or 558nm, respectively. Hydroxyproline content was expressed as μg hydroxyproline/mg muscle wet weight.

### Cardiomyocyte size determination

Hematoxylin-eosin (H&E) stained short-axis tissue sections of 10-μm thickness were digitized using light microscopy (x600 magnification; Nikon Eclipse E600). Myocytes with myofilaments surrounding the nucleus were identified separately from the left ventricular free wall and interventricular septum (minimum 50 myocytes; mean ± SEM—60.8±9.3). Myocyte diameter was determined as the minimum transverse diameter at the level of the nucleus (Image Pro Plus analysis software; Media Cybernetics) [26].

### Statistical analysis

Statistical analysis was performed using computer software, Statistical Package for Service Solution (SPSS 20.0). Normality of the data for different parameters was determined. Two-way ANOVA was used for determining the main effect and interaction between two variables (Age and Exposure). Differences between the groups were compared using an independent t-test for normally distributed data or Mann-Whitney U-test for data that did not meet assumptions for normality. To compare differences for repeated measures (ventilatory parameters) within the group, paired t-test was used. Statistical significance was set at p<0.05. All the values in the text, tables and figures are presented as Mean ± SEM.

## Results

### Body weight, Heart weight and Hematocrit

Body weights at baseline and at the end of experimental period are shown in Table 1. At baseline, no significant difference was noted in body weights of *mdx* mice across all the study groups. At the end of the 12 weeks experimental period, EH exposed mice from both the 6-mo and the 18-mo groups weighed significantly less as compared to their RA age matched controls. Consistent with previous studies, over the course of study, EH exposed *mdx* mice in both age groups did not exhibit weight gain. Instead, EH exposure led to decreases in body weight in both 6-mo and 18-mo old mice (3% and 17% respectively). As stated previously, food and water were available ad libitum during all phases of the study, even during EH exposure.

**Table 1:** Effect of 12 weeks of episodic hypoxia (EH) on body weight, hematocrit and heart weight in both 6-mo and 18-mo old *mdx* mice.

	6-mo RA	6-mo EH	18-mo RA	18-mo EH
	(n = 22)	(n = 18)	(n = 23)	(n = 12)
**Initial Body Weight, g**	34.9 ± 0.6	35.5 ± 0.7	36.9 ± 0.6	35.5 ± 0.5
**Final Body Weight, g**	38.1 ± 0.8	34.5 ± 0.6 ^b^	35.9 ± 0.8	29.4 ± 0.5 ^b^
**Hematocrit, %**	44.2 ± 0.5	46.1 ± 0.4 ^b^	42.6 ± 0.7	43.9 ± 0.7
**Heart Weight, mg**	149.2 ± 2.6	142.0 ± 3.0	157.7 ± 3.5 ^a^	148.3 ± 5.6
**Heart weight/Final Body Weight, mg/g**	3.9 ± 0.1	4.1 ± 0.1 ^b^	4.4 ± 0.1 ^a^	5.1 ± 0.2 ^b^

Values represent Mean ± SEM; n, number of mice.

^**a**^ significant difference (p<0.05) 6-mo vs 18-mo old RA *mdx* mice.

^**b**^ significant difference (p<0.05) RA vs EH exposed mice of same age.

As noted previously in non-DMD mice [27, 28], Hct levels were significantly higher in EH exposed 6-mo old *mdx* mice compared to their age matched RA controls (Table 1). In contrast, Hct levels in EH exposed 18-mo old *mdx* mice were similar to levels noted in age matched RA mice (Table 1).

As shown in Table 1, 18-mo old RA mice showed significantly higher absolute as well as relative (heart weight/body weight) heart weight compared to 6-mo old RA mice. Consistent with previous findings [27], relative heart weights (heart weight/body weight) were noticed to be significantly higher in EH exposed mice as compared to their RA counterparts in both age groups.

### Cardiac function

#### Echocardiography

**Fig 2:**
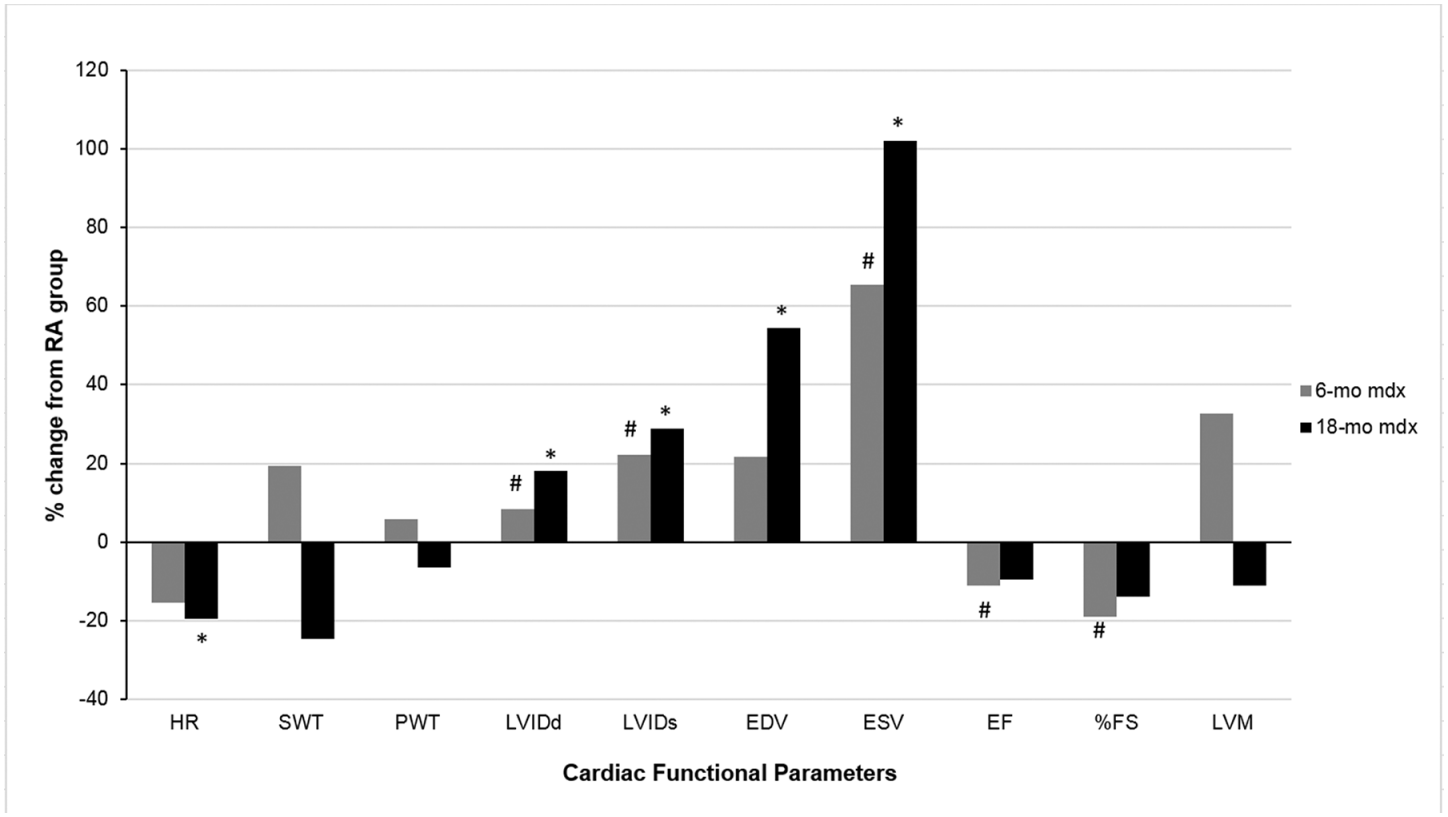
Effect of episodic hypoxia (EH) exposure on left ventricular functional parameters. The values are represented as percent change in LV parameter from RA exposed mice recorded by M-mode echocardiogram in 6-mo and 18-mo old *mdx* mice. *, indicates significant change (p<0.05) in cardiac functional parameters of 18-mo old *mdx* mice from their respective RA controls. #, indicates significant change (p<0.05) in cardiac functional parameters of 6-mo old *mdx* mice from their respective RA controls.

Echocardiographic analysis of cardiac function, especially LV function, is represented in Fig 2, and the data are shown in Table 2. RA exposed *mdx* mice in 6 and 18-mo old groups did not show any significant difference in HR at rest under anesthesia. However, EH exposed *mdx* mice in both age groups exhibited significantly lower HR than their age matched RA controls.

**Table 2:** Left Ventricular parameters measured by M-mode echocardiogram in 6-mo and 18-mo old *mdx* mice exposed to room air (RA) and episodic hypoxia (EH).

		6-mo RA	6-mo EH	18-mo RA	18-mo EH
		(n = 7)	(n = 7)	(n = 9)	(n = 5)
**HR**	bpm	330 ± 14	279 ± 11 ^b^	310 ± 20	250 ± 13 ^b^
**SWT**	mm	1.47 ± 0.12	1.75 ± 0.27	2.01 ± 0.20 ^a^	1.51 ± 0.18
**PWT**	mm	1.49 ± 0.14	1.58 ± 0.09	1.58 ± 0.08	1.48 ± 0.11
**LVIDd**	mm	2.60 ± 0.07	2.82 ± 0.06 ^b^	2.50 ± 0.13	2.95 ± 0.10 ^b^
**LVIDs**	mm	1.48 ± 0.12	1.82 ± 0.08 ^b^	1.50 ± 0.11	1.94 ± 0.17 ^b^
**EDV**	ml	0.047 ± 0.004	0.057 ± 0.005	0.044 ± 0.007	0.068 ± 0.007 ^b^
**ESV**	ml	0.010 ± 0.002	0.017 ± 0.002 ^b^	0.011 ± 0.002	0.021 ± 0.005 ^b^
**EF**	%	79.2 ± 3.3	70.5 ± 2.2 ^b^	76.7 ± 2.7	69.5 ± 5.1
**%FS**	%	42.6 ± 3.1	34.6 ± 1.6 ^b^	40.1 ± 2.4	34.5 ± 4.1
**LVM**	mg	135 ± 20	179 ± 27	177 ± 15 ^a^	157 ± 19

Values represent means ± SEM. p< 0.05.

^**a**^ significant difference (p<0.05) 6-mo vs 18-mo old RA *mdx* mice.

^**b**^ significant difference (p<0.05) RA vs EH exposed *mdx* mice of same age.

HR, heart rate; SWT, septal wall thickness; PWT, posterior wall thickness; LVIDd, left ventricular internal dimension at end-diastole; LVIDs, left ventricular internal dimension at end-systole; EDV, end diastolic volume; ESV, end systolic volume; FS, fractional shortening; LVM, left ventricular mass.

There were no detectable differences in left ventricular dimensions (LVIDs, LVIDd), volumes (EDV, ESV) and contractility (EF and FS) between 6-mo and 18-mo old RA exposed *mdx* mice. However, 18-mo old RA *mdx* mice showed significantly thicker septal wall (SWT) and higher LV mass (LVM) compared to 6-mo old RA mice.

In both age groups, chronic EH exposure led to significant changes in LV dimension and function. A significant increase in LV dimension (LVIDs and LVIDd) was noted in EH exposed 6-mo and 18-mo old *mdx* mice compared to their age matched RA counterparts. In 18-mo EH mice, LV volume (ESV, EDV) were significantly higher than their RA controls. In 6-mo EH group, though ESV was significantly greater than 6-mo RA mice, EDV failed to show any significant difference. As expected, a significant decrease in LV contractility (EF, FS) was noted in 6-mo EH group compared to their age matched RA controls. However, EH exposed 18-mo old *mdx* mice failed to show any significant decrease in LV contractility than to their age matched RA exposed mice.

#### Histological Analysis

**Fig 3:**
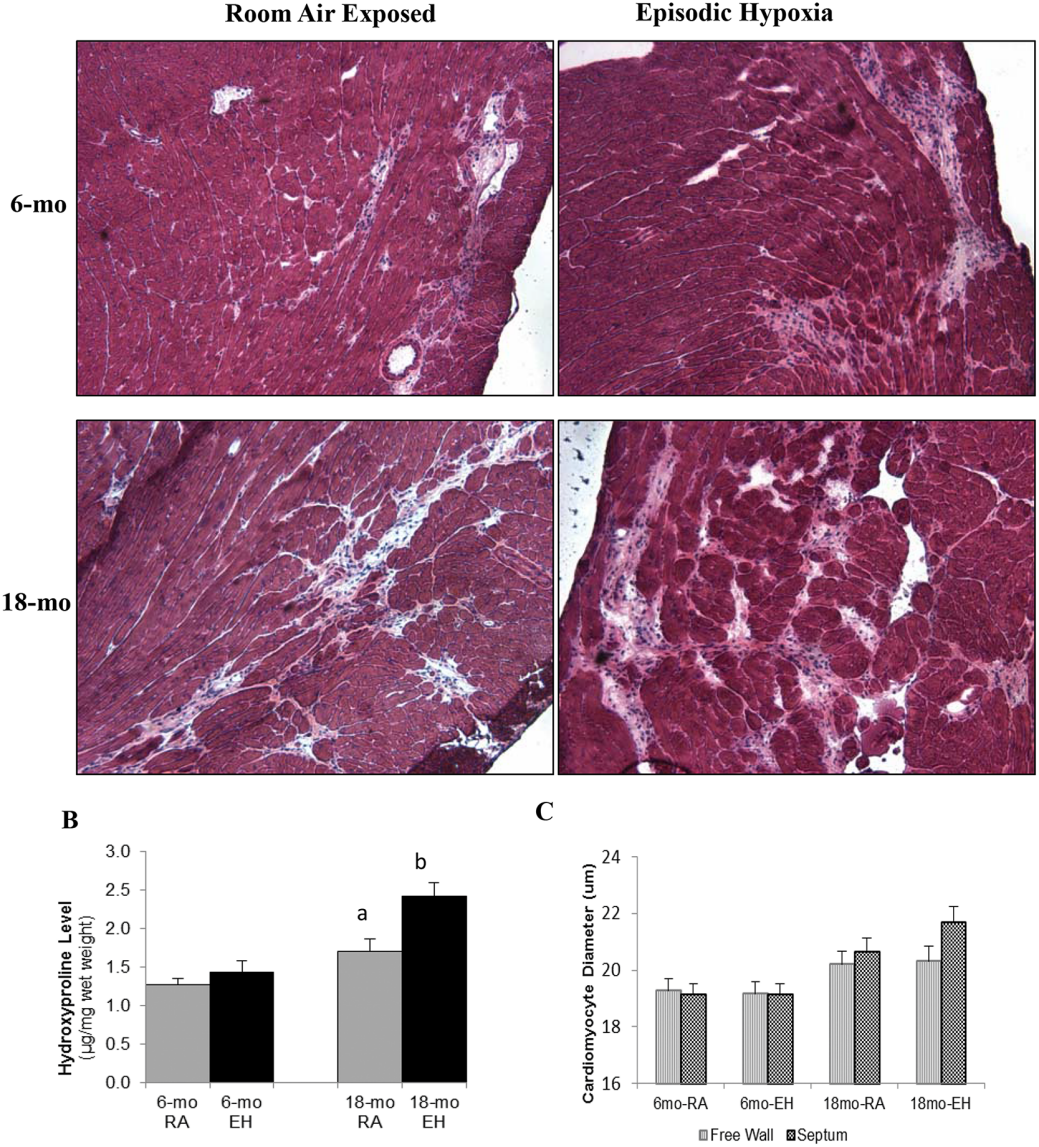
Histology of cardiac muscle and hydroxyproline content. (A) Photomicrograph of cardiac muscle (H&E stained, 10x) from 6-mo and 18-mo old mdx mice exposed to room air (RA) and episodic hypoxia (EH). Note higher amount of fibrosis and interstitial space in 18-mo old *mdx* mice as compared to 6-mo old *mdx* mice. EH exposed mice heart showed higher amount of fibrosis and interstitial space as compared to RA controls in 18-mo old *mdx* mice. (B) Cardiac hydroxyproline levels (μg/mg wet tissue weight) measured by biochemical analysis. a, significant difference (p<0.05) 6-mo vs 18-mo old RA *mdx* mice; b, significant difference (p<0.05) RA vs EH group of same age. (C) Cardiomyocyte Size, Effect of aging and episodic hypoxia on cardiomyocyte (LV free wall and septal) size in mdx mice.

Representative images of LV sections from each group are shown in Fig 3A. Cardiomyocyte diameter from both free LV wall and septal wall was determined (Fig 3C). EH exposure did not result in any significant difference in cardiomyocyte size in both 6-mo and 18-mo old *mdx* mice (Table 3).

**Table 3:** Histological parameters of diaphragm and cardiac muscle.

	6-mo RA	6-mo EH	18-mo RA	18-mo EH
**Cardiac Muscle**				
Free Wall Cardiomyocyte diameter (μm)	19.3 ± 0.4	19.2 ± 0.4	20.2 ± 0.5	20.3 ± 0.5
Septal Wall Cardiomyocyte diameter (μm)	19.1 ± 0.4	19.1 ± 0.4	20.6 ± 0.5	21.7 ± 0.6
**Diaphragm Muscle**				
Thickness, μm	586 ± 25	622 ± 23	415 ± 25 ^a^	350 ± 16 ^b^
Interstitial Space, %	17.5 ± 0.6	17.1 ± 0.9	32.1 ± 1.1 ^a^	35.9 ± 1.4 ^b^
Central Nucleated Fibers, %	27 ± 2	30 ± 2	11 ± 1 ^a^	13 ± 1

Values represent Mean ± SEM; n, number of mice.

^**a**^ indicates significant difference(p<0.05) 6-mo vs 18-mo old RA *mdx* mice.

^**b**^ indicates significant difference (p<0.05) RA vs EH group of same age.

#### Hydroxyproline Analysis

The hydroxyproline levels in 18-mo old *mdx* mice heart tissue were significantly higher (~ 34%) as compared to 6-mo old *mdx* mice heart in both EH and RA groups (Fig 3B).

In 6-mo old *mdx* mice, EH exposure did not lead to changes in heart hydroxyproline levels (1.27 ± 0.08 vs 1.42 ± 0.15 μg/mg wet weight, RA and EH respectively, NS). In contrast, EH exposure in 18-mo old mice was associated with an elevation in heart hydroxyproline level (1.70 ± 0.15 vs 2.42 ± 0.17 μg/mg wet weight, P<0.05).

### Respiratory function

#### Ventilation

As a comprehensive global assessment of ventilatory function, breathing patterns at rest and in response to hypercapnic challenge (4% CO_2_) were measured in awake mice. The data are shown in Table 4. At rest (Table 4), we observed that 18-mo-RA animals had a significantly different ventilatory profile than 6-mo old mice. Overall, exposure to EH in both 6-mo and 18-mo old mice did not significantly affect resting ventilatory patterns.

**Table 4:** Ventilatory parameters at rest and during hypercapnic exposures in 6-mo and 18-mo old *mdx* mice exposed to RA and EH.

	6-mo RA	6-mo EH	18-mo RA	18-mo EH
	(n = 8)	(n = 8)	(n = 8)	(n = 8)
**Normoxia**				
F, breaths/min	200 ± 10	200 ± 8	246 ± 4 ^a^	223 ± 8 ^b^
V_T_, ml/kg	1.52 ± 0.08	1.49 ± 0.11	1.52 ± 0.08	1.57 ± 0.07
V_E_, ml/kg/min	0.31 ± 0.03	0.30 ± 0.02	0.37 ± 0.02 ^a^	0.35 ± 0.02
**Hypercapnia (4% CO_2_)**				
F, breaths/min	258 ± 5 ^c^	239± 7 ^b^^c^	254 ± 3	224 ± 7 ^b^
V_T_, ml/kg	2.23 ± 0.11 ^c^	2.35 ± 0.13 ^c^	1.96 ± 0.15 ^c^	1.70 ± 0.06
V_E_, ml/kg/min	0.58 ± 0.03 ^c^	0.57 ± 0.05 ^c^	0.50 ± 0.04 ^c^	0.38 ± 0.02 ^b^

Values represent Mean ± SEM; n, number of mice.

^**a**^ significant difference (p<0.05) 6-mo vs 18-mo old RA *mdx* mice.

^**b**^ significant difference (p<0.05) RA vs EH exposed mice of same age.

^**c**^ significant difference (p<0.05) normoxia vs hypercapnia in all the groups

F, frequency; V_T_, tidal volume; V_E_, minute ventilation

In response to hypercapnia, 6-mo old *mdx* mice from both RA and EH groups showed significant increases in ventilation (f, V_T_, and V_E_) over their resting RA values (Table 4). Although, 18-mo old RA mice increased ventilation in response to hypercapnia over their resting values, the response was much less than that observed in 6-mo old mice. In sharp contrast, hypercapnia failed to elicit any ventilatory response in 18-mo EH mice above their resting values (Table 4).

#### Diaphragm Contractility

**Fig 4:**
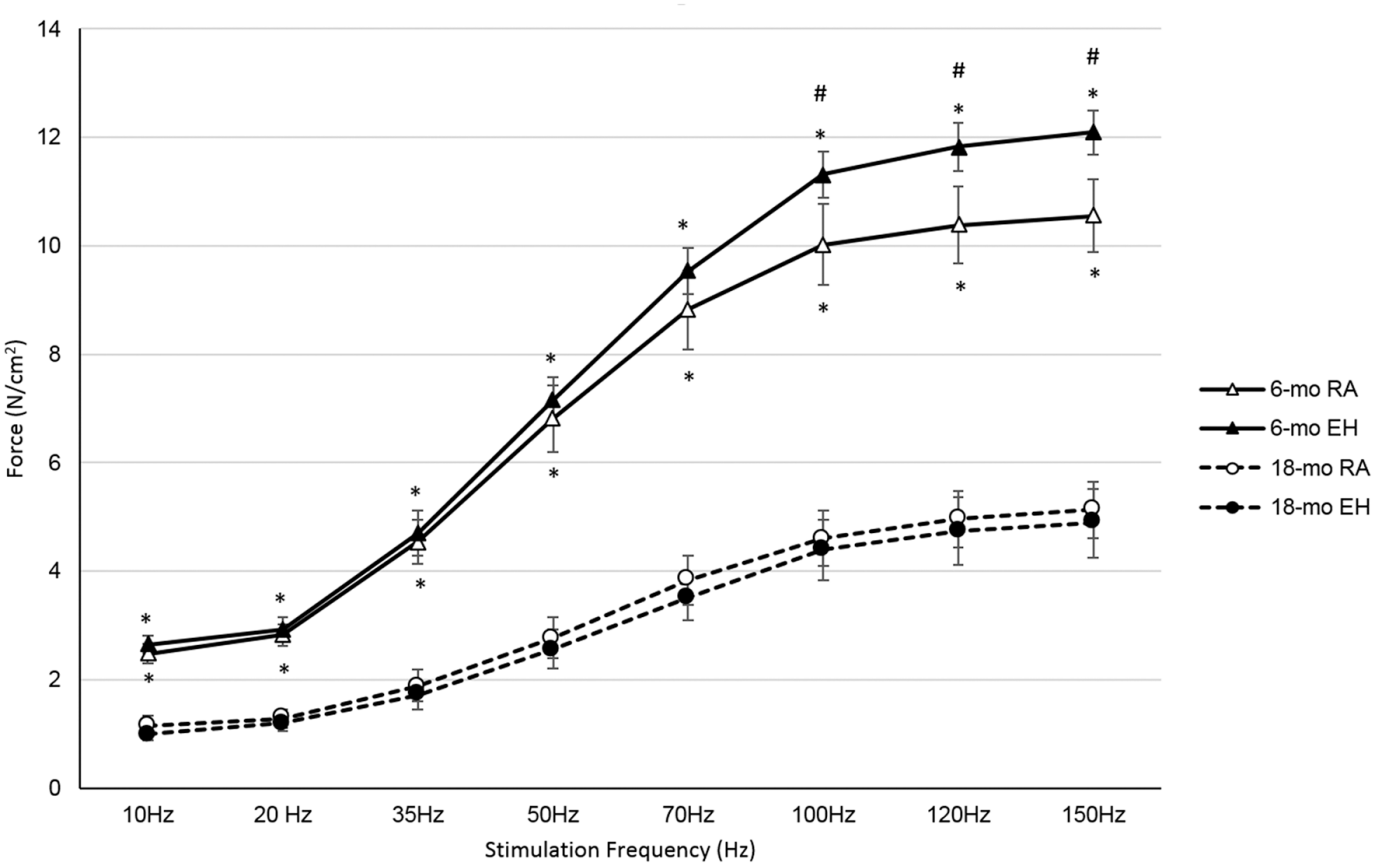
Force-frequency profile of diaphragm from 6-mo and 18-mo old mdx mice exposed to room air (RA) and episodic hypoxia (EH). At all stimulation frequencies diaphragm forces were significantly lower in 18-mo old *mdx* mice in both RA and EH groups. 6-mo old *mdx* mice exposed to EH showed higher diaphragm forces compared to RA group at higher stimulation frequencies, but in 18-mo old *mdx* mice diaphragm forces were not significantly different in RA and EH groups. *, indicates significant difference between 6-mo RA vs 18-mo RA and 6-mo EH vs 18-mo EH. #, indicates significant difference between 6-mo RA vs 6-mo EH.

In order to assess respiratory muscle function, force output of the isolated diaphragm (primary respiratory muscle) bundles were measured. Intrinsic diaphragm forces corrected for cross sectional area were significantly reduced in 18-mo old *mdx* mice as compared to 6-mo old *mdx* mice across all the stimulation frequencies (Table 5 and Fig 4). On average, maximal tetanic force for 18-mo old RA *mdx* mice was ~50% lower than that generated by 6-mo old RA mice.

**Table 5:** In-vitro mechanical characteristics of diaphragm muscle of 6-mo and 18-mo old *mdx* mice exposed to RA and EH for 12 weeks.

		6-mo RA	6-mo EH	18-mo RA	18-mo EH
		(n = 10)	(n = 9)	(n = 9)	(n = 10)
**TPT**	(msec)	20.1 ± 0.3	20.1 ± 0.5	19.6 ± 0.3	21.8 ± 0.9 ^b^
**1/2 RT**	(msec)	20.1 ± 0.9	17.3 ± 1.0 ^b^	19.8 ± 1.2	20.4 ± 1.8
**Pt**	(N/cm^2^)	2.26 ± 0.17	2.44 ± 0.15	0.91 ± 0.07 ^a^	0.83 ± 0.11
**Po**	(N/cm^2^)	10.6 ± 0.7	12.1 ± 0.4 ^b^	5.1 ± 0.5 ^a^	4.9 ± 0.6
**Lo**	(cm)	0.94 ± 0.03	0.96 ± 0.02	0.92 ± 0.03	0.92 ± 0.02
**Pt/Po**		0.22 ± 0.02	0.20 ± 0.01	0.17 ± 0.02 ^a^	0.18 ± 0.01

Values represent Mean ± SEM; n, number of mice

^**a**^ indicates significant difference(p<0.05) 6-mo vs 18-mo old RA *mdx* mice.

^**b**^ indicates significant difference (p<0.05) RA vs EH group of same age.

TPT, time to peak twitch tension; ½ RT, half relaxation time; Pt, peak twitch force; Po, maximal isometric force; Lo, optimal muscle length.

In response to EH exposure, diaphragms from 6-mo mice generated significantly higher forces (~14%) compared to forces recorded in 6-mo old RA mice (Table 5). In contrast, diaphragm force output was similar in RA and EH exposed 18-mo old *mdx* mice (Fig 4).

#### Histological analysis

**Fig 5:**
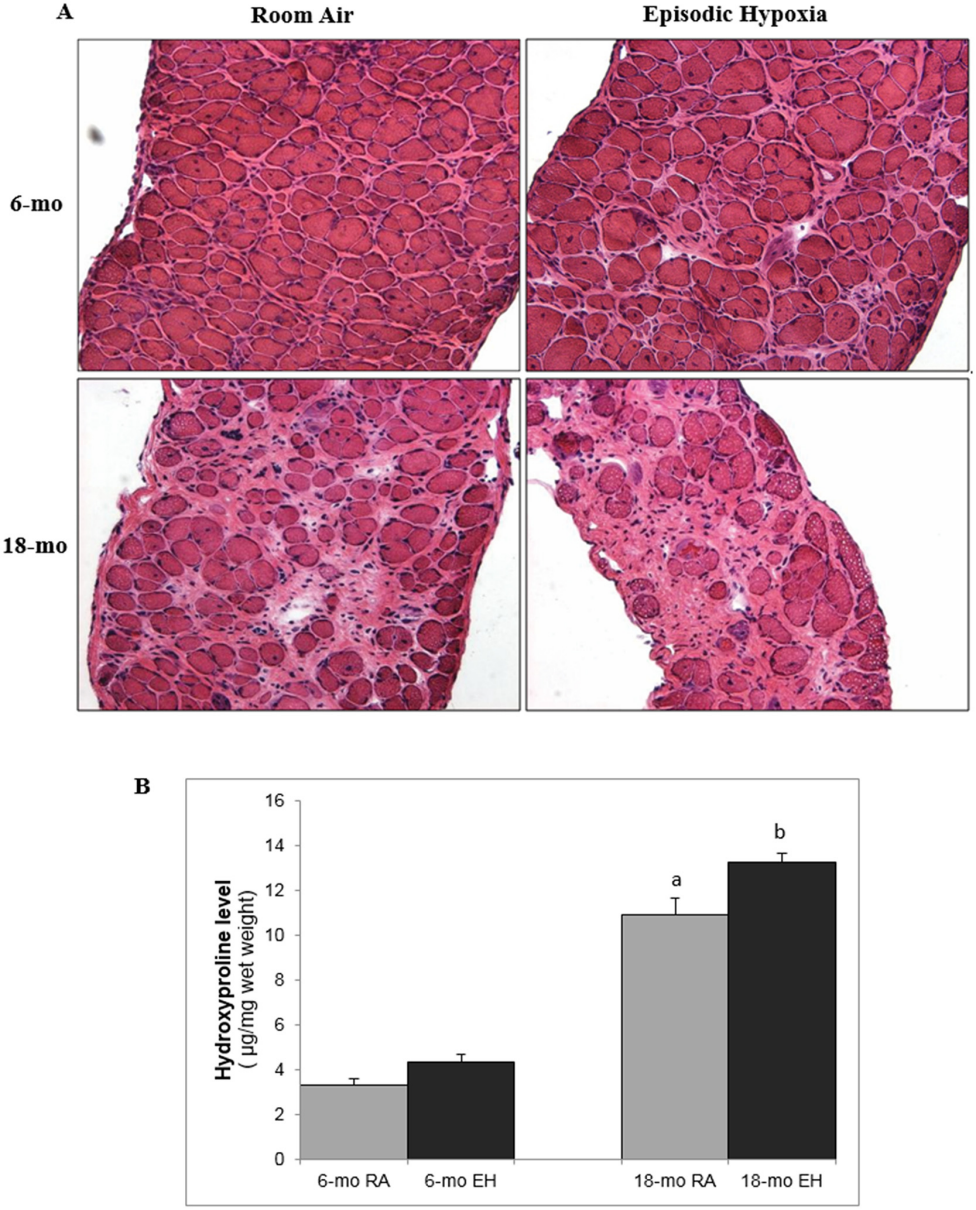
Histology of diaphragm muscle and hydroxyproline content. (A) Photomicrograph of diaphragm muscle (H&E stained, 20x) from 6-mo and 18-mo old mdx mice exposed to room air (RA) and episodic hypoxia (EH). Note higher amount of fibrosis and interstitial space in 18-mo old *mdx* mice as compared to 6-mo old *mdx* mice. EH exposed mice diaphragm showed higher amount of fibrosis and interstitial space as compared to RA controls in both 6-mo and 18-mo old *mdx* mice. Difference in diaphragm thickness as well as presence of muscle fibers with central nuclei can also be noticed in this image. (B) Diaphragm hydroxyproline levels (μg/mg wet tissue weight) measured by biochemical analysis. a, significant difference(p<0.05) 6-mo vs 18-mo old RA *mdx* mice; b, significant difference (p<0.05) RA vs EH group of same age.

In order to assess structural changes at the cellular level, we analyzed histological parameters in both diaphragm and cardiac muscles. Representative images of diaphragm sections from each group are shown in Fig 5A. Histological data are shown in Table 3. The diaphragms from 18-mo old *mdx* mice were significantly thinner (~30%), with increased interstitial space (~80%) as compared to 6-mo old *mdx* mice.

In 6-mo old mice, EH exposure had no impact on diaphragm thickness and interstitial space. In contrast, EH exposure in 18-mo old mice led to significant decreases in diaphragm thickness (~16%) and to a concomitant increase in interstitial space (~12%).

Central nucleation, an indicator of regeneration process in muscle fiber after degeneration or damage [29], was measured (Table 3). Diaphragm muscle fibers of 6-mo old *mdx* mice in both RA and EH group showed significantly higher (245% and 230% respectively) number of centrally nucleated fibers than those noted in 18-mo old *mdx* mice (Table 3). EH exposure had no impact on central nucleation in either 6-mo old or 18-mo old *mdx* mice.

#### Hydroxyproline Analysis

Hydroxyproline content, an index of collagen content and fibrosis in muscle, was also measured. Diaphragms from 18-mo old RA *mdx* mice revealed significantly higher hydroxyproline levels (~230%) as compared to 6-mo old RA mice (Fig 5B). There was no significant difference in diaphragm hydroxyproline levels between RA and EH exposed 6-mo old *mdx* mice (3.3 ± 0.3 vs 4.4 ± 0.4 μg/mg wet weights respectively, N. S.), while EH exposure in18-mo old *mdx* mice led to significantly higher hydroxyproline level compared to age matched RA controls (13.3 ± 0.4 vs 10.9 ± 0.8 μg/mg wet weights respectively, p<0.05).

## Discussion

To our knowledge, this is the first comprehensive study to investigate the combined impact of EH and aging on cardiac and respiratory function in the dystrophin-deficient *mdx* mice. The *mdx* mouse has been validated as a model for DMD and has been studied extensively for several decades. Although limb muscles of *mdx* mice do not show the same extent of pathology or degeneration as seen in DMD [11], the diaphragm muscle has been shown to closely mirror the pathology and degeneration-regeneration pattern noted in skeletal muscles of DMD patients [30]. In addition, the cardiac muscles of the *mdx* mouse also present with a similar pathological profile as seen in DMD [13].

Dysfunctions of the respiratory and cardiac systems primarily occur at different stages in the course of DMD. Respiratory abnormalities are usually noted first, at around 8–10 years of age, while cardiac abnormalities in DMD are usually detected at about 18 years of age [31]. Similarly, in *mdx* mice the respiratory abnormalities are diagnosed prior to detecting cardiac dysfunction. The respiratory muscle dysfunction, including that of the diaphragm, begins at 3–4 weeks of age in *mdx* mice and progresses as the animal age, while cardiomyopathy in *mdx* mice is detected usually after 9 months of age [13, 32]. In the current study, 3-mo old *mdx* mice were selected to represent a younger stage where respiratory but not cardiac dysfunction would be expected, and 15-mo old *mdx* mice represented an older group, where both cardiac and respiratory dysfunction would be present simultaneously.

SBD and sleep apnea are common findings in DMD. Indeed, over 80% patients show disturbed breathing during sleep with gas exchange abnormalities and episodes of hypopnea and apnea. Smith et al. (1988) reported that patients of DMD undergo repeated bouts of hypoxia during sleep, during which SaO2 levels falls from baseline of 95.4 +/- 0.6% (SEM) to a mean nadir of 74.2 +/- 3.9% [7]. Since sleep apnea patients experience similar repeated bouts hypoxemia during sleep, experimentally induced intermittent hypoxia/episodic hypoxia have been used in animal models to simulate sleep apnea. To validate whether our EH model induced apneic events that were akin to those previously reported in DMD, we conducted an initial pilot study to assess SaO2 levels (MouseOx, Starr Life Sciences, Oakmont, PA) during EH in awake *mdx* mice. In five 4-mo old *mdx* mice, we determined that SaO_2_ during the EH as selected for the current study (21% to 6% FiO_2_), fluctuated between 97.2 ± 1.7% during RA and reached a nadir of 71.84 ± 1.8% after 90 sec of the hypoxic exposure (unpublished observation). Thus, the EH exposure used in the current study closely mimicked findings as reported in DMD patients.

### Effect of EH on body weight and hematocrit

Consistent with previous EH studies in non-DMD rodents [27, 28], *mdx* mice exposed to EH of in 6-mo old groups similarly responded with changes in body weight and Hct levels (Table 1). However, 18-mo old *mdx* mice did not show significant elevations in Hct level on EH exposure, suggesting failed compensation. EH increases hypoxic stress which results in a decrease in body weight of mice over the experimental period. Long term hypoxia exposure normally leads to activation of hypoxia inducible factor (HIF) 1-α, which in turn causes increased production of erythropoietin[33, 34]. Erythropoietin subsequently increases red blood cell production leading to elevated Hct level [35]. The impact on weight and Hct levels support that EH exposed *mdx* mice of both age groups received a hypoxic stimulus that was physiologically challenging.

### Cardiac function

In DMD patients, cardiac muscles initially undergo hypertrophy due to muscle damage. With increasing age, damaged cardiac tissue undergoes fibrosis, necrosis and loss of muscle fibers, ultimately leading to dilated cardiomyopathy [36]. In DMD patients, left ventricular wall initially shows signs of myocardial fibrosis which then spread to the interventricular septum and subsequently to the right ventricle [13]. Similar cardiac pathology has been reported to occur in *mdx* mice [14]. Chronic exposure to sleep apnea causes an increase in systemic blood pressure and later it can result in left ventricular hypertrophy and cardiomyopathy [10]. To our knowledge, no study has investigated the impact of EH on cardiac dysfunction in *mdx* mice. This is not trivial, as some studies have reported that more than 80% of DMD patients suffer from sleep disturbed breathing with significant nocturnal desaturation [5].

In this study, we observed decreased heart rate at rest in EH exposed mice as compared to their RA controls in both 6-mo and 18-mo old *mdx* mice. However, as Ketamine/Xylazine anesthesia is known to have negative ionotropic effect, the results may have been confounded even at similar dose of anesthetic. Although some studies have shown increase in heart rate after exposure to hypoxia in non-DMD mice due to stimulation of sympathetic system [15, 37], the results are inconsistent, as other studies, consistent with our findings, have reported decreased heart rate in awake mice after hypoxic exposure [38].

Consistent with pathophysiology of cardiomyopathy in DMD and *mdx* mice [39–41], we expected more severe cardiac dysfunction in our 18-mo old group as compared to 6-mo old group. Although older mice had significantly thicker septal wall and higher LV mass than 6-mo old mice, LV contractility was noted not to be significantly different. In contrast to previous studies, which reported decrease in EF and FS with aging [42, 43], we did not see significant difference in EF and FS in both age groups. One possible explanation is the development of cardiac dysfunction by 6 month of age in *mdx* mice, which therefore is not significantly different than older 18 month *mdx* mice. With 12 weeks of EH exposure, no significant difference was noted in LVM and SWT in both age group mice. Even though, not significantly different, once cannot ignore opposite trend in both LVM and SWT parameters as a result of 12 weeks EH exposure in both age groups. These findings suggest that 12 weeks EH exposure results in hypertrophic changes in 6-mo old mice and increased muscle necrosis with muscle fiber loss in older 18-mo *mdx* mice.

Contrary to a previous studies [13, 39], who failed to detect cardiomyopathy using non-invasive techniques (echocardiogram) before 7–8 months of age in *mdx* mice, we found that if *mdx* mice are exposed to episodic hypoxia, detectable cardiomyopathy can be detected at an early age in *mdx* mice. Danialou et al., 2001 [44] reported that *mdx* hearts are susceptible to damage under mechanical stress and reported abnormal cardiac function using invasive techniques. Our findings support that increased stress, in the form of EH exposure, in 6-mo old *mdx* mice results in decreased LV contractility. It was interesting to see early signs of dilated cardiomyopathy (increased LVID) at this age with EH exposure, as dilated cardiomyopathy is usually seen in later stages of life in both *mdx* mice as well as DMD patients [31, 45]. EH exposed 18-mo old *mdx* mice also showed signs of dilated cardiomyopathy which is consistent with previous studies [13].

As shown previously in wild-type and *mdx* mice [13], we noted increases in hydroxyproline levels, an indicator of cardiac fibrosis, with aging (Fig 3B). Signs of increased myocardial fibrosis with mononuclear infiltrates were also evident on histological examination of heart tissue (Fig 3A). EH exposure in wild-type mice has also been shown to increase cardiac fibrosis [46]. In our study, although EH exposure led to increased hydroxyproline in 18-mo old mice, no significant increase in hydroxyproline level was noted in 6-mo old *mdx* mice, suggesting increased susceptibility of older cardiac muscle to damage in response to EH. Increase in fibrosis with aging has been reported previously in normal wild type as well as *mdx* mice [39, 47]. In older *mdx* mice, there are many factors that have been implicated in the increased fibrosis such as increased muscle injury-repair mechanism and increased TGF-β level [39, 48].

A previous study[13] has shown cardiomyocyte hypertrophy with aging in *mdx* mice. In our study, we did not see significant difference in cardiomyocyte diameter between 6-mo and 18-mo *mdx* mice. Cardiomyocyte diameter tended to be larger in 18-mo *mdx* mice as compared to 6-mo old mice, but failed to reach significance (Table 3 and Fig 3C). We also found no significant effect of EH exposure on cardiomyocyte size in *mdx* mice. No study has reported the effect of EH in *mdx* mice. However, in non-DMD mice, several studies reported cardiomyocyte hypertrophy in response to EH exposure [49, 50], while others observed no hypertrophy with EH exposure [51]. No significant change seen in cardiomyocyte size in response to EH can be due to altered response to stress in these mice due to dystrophin deficiency.

There are at least two possible mechanisms, mechanical damage and oxidative stress, which may account for the cardiac dysfunction seen following EH exposure. Exposure to EH in non-DMD rodent models increases the mechanical stress on the heart resulting in hypertension [27, 38], decreased cardiac contractility [46] and ischemic events [52]. The lack of dystrophin makes cardiac muscle fibers more susceptible to membrane damage during mechanical stress. As a result, cardiac muscle membrane becomes more permeable to influx of Ca^2+^ [53, 54]. Ca^2+^ overload is associated with a cascade of events such as increased protease activity, impaired mitochondrial oxidative phosphorylation leading to cardiomyocyte necrosis, and ultimately to cell death [54]. In addition, sleep apnea as well as long term exposure to EH have been shown to be associated with myocardial ischemia [55–57]. Episodic hypoxia with its resultant fluctuations in SaO_2_ levels causes repeated episodes of hypoxia/reoxygenation, which leads to increase susceptibility of muscle to ischemia/reperfusion injury on chronic exposure [52]. The role of reactive oxygen species in ischemia/reperfusion injury has been well documented [58–60]. Thus, synergistic effect of mechanical instability and oxidative stress in *mdx* mice following EH may be singularly or partially responsible for the cardiac dysfunction that we observed. Additional studies will be required to investigate which of these two possible mechanisms play the major role in cardiac dysfunction.

### Respiratory function

Due to the absence of dystrophin, skeletal muscles in *mdx* mice are susceptible to injury induced by increased mechanical stress [2]. The loss of dystrophin makes the sarcolemma more fragile leading to abnormal calcium influx and recurrent cycles of myofiber degeneration-regeneration, thus leading to progressive muscle weakness [30, 53]. Along with increased susceptibility to muscle injury, these muscles also have impaired repair mechanism. In normal muscle repair, satellite cells play a major role in regeneration of muscle fibers [61]. In DMD and *mdx* skeletal muscle, however there is continuous degeneration-regeneration of muscle fibers, along with inflammation [62], and satellite cells lose their capacity over time for muscle repair [23]. Damaged muscle fibers are eventually replaced by fibrotic tissue leading to muscle dysfunction [48]. Consistent with previous findings, we noted that aging influenced diaphragmatic force [63, 64] (Fig 4), thickness, interstitial space [65], central nucleation [65], and hydroxyproline levels (Table 3 and Fig 5B).

On histological analysis, the higher number of muscle fibers exhibiting central nucleation in 6-mo old RA *mdx* mice supports elevated degeneration-regeneration process in diaphragm muscle fibers. In contrast, diaphragms of 18-mo old RA *mdx* mice exhibited significantly lower number of central nucleation suggesting satellite cells exhaustion and/or reduced regenerative capacity with ageing [66, 67]. Histological analysis of diaphragm muscle also revealed scattered areas of fibrosis consistent with our hydroxyproline analysis. Consistent with previous findings [11], 18-mo old diaphragm tissue demonstrated more areas of fibrosis and mononuclear infiltrates than noted at 6 months.

Relative force of isolated diaphragm corrected for cross sectional area, as shown in Fig 4, in reality underestimates the effect of age on in-situ maximal force generation. Since the diaphragm was not only noted to be thinner in older RA mice (Table 3), but that a larger fraction of the diaphragm was occupied by non-muscular tissues (Fig 5A), we can estimate that absolute peak diaphragm forces that could be generated in-situ (assuming equal neural activation) would be 28% lower in older RA *mdx* mice as compared to their younger counterparts. In contrast, 18-mo old EH exposed *mdx* mice showed no change in relative diaphragm strength (Fig 4). However, since these mice also demonstrated significantly thinner diaphragms (Table 3) with larger fraction of cross-section occupied by fibrotic tissue (Table 3), one would predict that in-situ diaphragm forces in 18-mo EH mice would be further compromised by 20%.

Previously, we reported [68] that 12 weeks of EH exposure in 6-mo old *mdx* mice resulted in a reduction in relative diaphragm force by about 30%. In sharp contrast, relative diaphragm strength in the current study in 6-month old *mdx* mice increased (Table 5) following 12 weeks of EH exposure (Fig 4).The divergent responses to EH between our current and our former study is likely attributed to the hypoxic stress levels imposed and the resultant physiological responses on the respiratory system. One would expect that, more severe hypoxia would result in a more robust ventilatory response (diaphragm activation) during EH. The severity of whole body activity levels have been reported to result in contrasting findings on limb muscles strength. Voluntary activity in *mdx* mice appears to have a beneficial effect on limb muscle strength [69–71], whereas forced activity at higher intensities (and muscle recruitment) has been shown to be detrimental and to enhance weakness of limb muscles [72, 73]. Thus, a similar conclusion can be drawn here for diaphragm strength, in that respiratory muscle recruitment at less severe levels can lead to improvements in diaphragm strength, whereas more severe recruitment and higher stress can lead to reductions in diaphragm strength.

Ventilatory parameters were recorded using modified barometric plethysmography in all *mdx* mice at rest and in response to hypercapnia (4% CO_2_). Consistent with a previous study [65], older, 18-mo RA *mdx* mice showed higher frequency of breathing compared to younger, 6-mo RA mice. Since tidal volume between these groups were similar, ventilation was noted to be higher in older *mdx* mice. Aging, as has been previously reported [65], was associated with an increase in ventilatory dysfunction, as 18-mo old *mdx* mice showed decreased response to 4% CO_2_ exposure as compared to 6-mo old *mdx* mice. Consistent with our diaphragm strength data (Fig 4) and the implications to in-situ diaphragm force discussed above, ventilatory function in awake mice showed no significant effect in ventilatory parameters due to EH exposure in 6-mo old *mdx* mice (Table 4). In contrast, 18-mo old *mdx* mice exposed to EH show considerable ventilatory dysfunction and in addition, failed to increase ventilation above resting room air baseline values in response to the hypercapnic challenge. As this effect appears to be focused on the control of respiration, rather than the diaphragm muscle itself, ventilatory dysfunction in18-mo EH *mdx* mice is most likely secondary to reduced ventilatory reserve, or to a severely blunted CO_2_ response. Indeed, a recent study has shown decreased peripheral chemosensory response and carotid body dysfunction in *mdx* mice [74]. Additional studies are needed to determine if carotid body dysfunction is exacerbated by sleep apnea in DMD.

There are several limitations of the current study design. One relates to the absence of wild type non-DMD mice. As our intent was to investigate the functional impact of EH on cardiac and respiratory function in dystrophin deficient mice, we purposely restricted our study to *mdx* mice. In the absence of normal wild type mice, we cannot address whether EH causes more or less severe cardiac and respiratory dysfunction in *mdx* mice. Although not a goal of our study, additional research is required to study this comparison. Another limitation is related to the use of anesthesia for the assessment of cardiac function. Ketamine/xylazine anesthesia is known to exert negative chronotropic and ionotropic effects in mice [75, 76]. For this reason, our original intent was to evaluate cardiac function in awake mice, and initial attempts were successful in all groups with the exception of the 18-mo old EH group. Since the first two 18-mo EH awake mice died during echocardiography and as we could ill afford to lose additional 18-mo EH mice during the procedure, we elected to study all animals under anesthesia. Also, we limited our analysis to LV function. Echocardiographic assessment of RV dimension and function is very challenging, especially in murine models due to their small size, retrosternal position and irregular shape. As technical limitation preclude accurate measurement of RV function, we decided to focus on LV function.

In summary, this is the first comprehensive study to report the effect of experimentally induced sleep apnea on cardiac and respiratory functions in DMD mice. Our results support that EH induces cardiac and respiratory dysfunction in *mdx* mice. In addition, the observed dysfunctions were more severe in older *mdx* mice. Our findings also highlight that EH can have divergent effects on the cardiac and respiratory systems. Indeed, in younger animals, EH produced improvement in isolated diaphragmatic function, while producing mild cardiac dysfunction. In older animals, EH proved to be detrimental to both the cardiac and respiratory systems. The results of the present study have important implications in DMD patients. As previously mentioned, over 80% of DMD patients suffer from sleep disordered breathing and a majority of these patients succumb to respiratory or cardiac failure. Although, nocturnal assisted ventilation is often employed in DMD patients, it is generally initiated during the late ambulatory or non-ambulatory stages, only after a positive diagnosis of hypoventilation or hypercapnia [4]. It is still debatable when to initiate nocturnal assisted ventilation to achieve maximum benefit. Based on the current findings, DMD patients may certainly benefit by earlier screening and interventions, especially since nocturnal hypoventilation is often detected in DMD patients presenting with normal daytime ventilation and oxygen saturation [7]. In addition, future studies are needed to investigate the putative mechanisms responsible for the respiratory and cardiac dysfunction and to study whether these deficits, once detected, can be reversed with effective treatment.
